# Readmission of older acutely admitted medical patients after short-term admissions in Denmark: a nationwide cohort study

**DOI:** 10.1186/s12877-020-01599-4

**Published:** 2020-06-11

**Authors:** M. Klinge, M. Aasbrenn, B. Öztürk, C. F. Christiansen, C. Suetta, E. Pressel, F. E. Nielsen

**Affiliations:** 1 Geriatric Research Unit, Department of Geriatrics, Copenhagen University Hospital, Bispebjerg and Frederiksberg, Denmark; 2 Novo Nordisk Foundation Center for Basic Metabolic Research, Faculty of Health and Medical Sciences, University of Copenhagen, Copenhagen, Denmark; 3 Department of Clinical Epidemiology, Aarhus University Hospital, Aarhus, Denmark; 4 Geriatric Research Unit, Department of Medicine, Herlev-Gentofte Hospitals, Copenhagen, Denmark; 5 CopenAge – Copenhagen Center for Clinical Age Research, University of Copenhagen, Copenhagen, Denmark; 6 Department of Emergency Medicine, Copenhagen University Hospital, Bispebjerg and Frederiksberg, Denmark; 7 Department of Emergency Medicine, Slagelse Hospital, Bispebjerg and Frederiksberge, Denmark; 8 Copenhagen Center for Translational Research, Copenhagen University Hospital, Bispebjerg and Frederiksberg, Denmark

**Keywords:** Emergencies, Patient readmission, Comorbidity, Geriatrics, Patient discharge

## Abstract

**Background:**

Knowledge of unplanned readmission rates and prognostic factors for readmission among older people after early discharge from emergency departments is sparse. The aims of this study were to examine the unplanned readmission rate among older patients after short-term admission, and to examine risk factors for readmission including demographic factors, comorbidity and admission diagnoses.

**Methods:**

This cohort study included all medical patients aged ≥65 years acutely admitted to Danish hospitals between 1 January 2013 and 30 June 2014 and surviving a hospital stay of ≤24 h. Data on readmission within 30 days, comorbidity, demographic factors, discharge diagnoses and mortality were obtained from the Danish National Registry of Patients and the Danish Civil Registration System. We examined risk factors for readmission using a multivariable Cox regression to estimate adjusted hazard ratios (aHR) with 95% confidence intervals (CI) for readmission.

**Results:**

A total of 93,306 patients with a median age of 75 years were acutely admitted and discharged within 24 h, and 18,958 (20.3%; 95% CI 20.1 - 20.6%) were readmitted with a median time to readmission of 8 days (IQR 3 - 16 days). The majority were readmitted with a new diagnosis. Male sex (aHR 1.15; 1.11 - 1.18) and a Charlson Comorbidity Index ≥3 (aHR 2.28; 2.20 - 2.37) were associated with an increased risk of readmission. Discharge diagnoses associated with increased risk of readmission were heart failure (aHR 1.26; 1.12 - 1.41), chronic obstructive pulmonary disease (aHR 1.33; 1.25 - 1.43), dehydration (aHR 1.28; 1.17 - 1.39), constipation (aHR 1.26; 1.14 - 1.39), anemia (aHR 1.45; 1.38 - 1.54), pneumonia (aHR 1.15; 1.06 - 1.25), urinary tract infection (aHR 1.15; 1.07 - 1.24), suspicion of malignancy (aHR 1.51; 1.37 - 1.66), fever (aHR 1.52; 1.33 - 1.73) and abdominal pain (aHR 1.12; 1.05 - 1.19).

**Conclusions:**

One fifth of acutely admitted medical patients aged ≥65 were readmitted within 30 days after early discharge. Male gender, the burden of comorbidity and several primary discharge diagnoses were risk factors for readmission.

## Background

The incidence of acute diseases that traditionally have led to hospitalization is expected to increase during the next decades, partly due to a large increase in the population above 80 years [1, 2]. Efficiency in the health care system is therefore needed. This may include improvement of the discharge processes, preventive measures to reduce unplanned readmissions, increased activity in outpatient clinics, and reductions in the length of hospitalization. However, optimal discharge timing is known to be challenging [3, 4]. Medical and social issues may remain unsolved if a patient is discharged prematurely, and long stays are costly with risk of nosocomial infections and other adverse events [5, 6]. A study from the United States including more than four million medical patients of all ages found a lower readmission rate with shorter length of stays (LOS) [7]. Even though many patients are rapidly discharged, a concern has been raised, that premature discharge of the oldest patients may lead to an increase in readmissions [8]. Whether early discharge of older patients is associated with higher risks of readmission is uncertain, and previous studies have provided conflicting results [9–11]. 30-day readmission rates from 13 to 22% have been reported in observational studies of older patients admitted with medical diagnoses [9–13]. However, readmission among older patients after very early discharge from hospitals is sparsely described. The aims of this study were therefore to examine the unplanned readmission rate among medical patients older than 65 years who were discharged within 24 h after acute admissions to Danish hospitals and further to examine potential risk factors for readmission including demographic factors, comorbidity and discharge diagnoses.

## Methods

### Design and setting

We conducted this cohort study using the Danish National Registry of Patients (DNRP) [14] and the Danish Civil Registration System (CRS) [15]. By using the unique personal identification number, assigned to all Danish residents, individual-level linkage between the national administrative registers is possible.

The Danish healthcare system offers equal and universal access for all residents. Danish patients who are hospitalized acutely are either referred by general practitioners (GPs) or when urgent treatment is required the patients can arrive on their own or by ambulance to the hospital. Patient care outside office hours is provided by the GPs in regional clinics from which patients can be referred acutely to hospital. The emergency health care service in Denmark is carried out by 21 acute care hospitals [16]. Acutely admitted patients are with few exceptions admitted via one common emergency department (ED) entrance staffed with specialists on a 24-h basis [16]. After the initial treatment in the ED the patients were either discharged or transferred to short stay ED units or medical wards for further observation and treatment. Danish privately funded hospitals have no acute patient intake [16].

### Study population

We identified eligible patients in the DNRP using the administrative codes for acute and medical admission. It has previously been shown that the registration of acute admission among medical patients in the DNRP has high validity [17]. We registered all older (aged ≥65 years) patients with an acute medical hospital admission between 1 January 2013 and 30 June 2014 and a length of hospital stay of ≤24 h. We excluded patients with contacts to a hospital emergency room without an admission to an emergency department or medical ward, patients who died during the first 24 h during their hospital stay, and patients who were transferred to other hospitals.

### Data source

The DNRP provides nationwide registration of detailed administrative and clinical data [14]. The registry contains information on all hospital contacts in Denmark, surgical procedures, major treatments performed, one primary discharge diagnosis, and secondary inpatient and outpatient discharge diagnoses. The diagnoses in the registry are coded by use of the Danish version of the International Classification of Diseases 10th edition (ICD-10).

Using the DNRP, we have characterized all patients by age and gender. For the index admission and the readmission, we obtained data on the admission and discharge time, Charlson Comorbidity Index (CCI, [18], primary discharge diagnoses (“index diagnosis”, main reason for hospitalization), secondary diagnoses, malignancy, R- and Z-diagnoses. The R-diagnoses are used if symptoms, signs and abnormal clinical and laboratory findings are not elsewhere classified, and Z-diagnoses are used when a diagnosis has not yet been confirmed. From the CRS we obtained information on civil status at the index admission time and vital status during the study period.

### Comorbidity

We have assessed the comorbidity burden by use of CCI, which is computed based on all primary and secondary discharge diagnoses that have been registered in the DNRP since 1977. The CCI score was divided into three levels: low (score 0), moderate (score 1-2), and high (score 3+) [19].

### Outcome

The primary endpoint was acute medical readmission (time to readmission) within the first 30 days of discharge after the index hospitalization.

### Statistical analysis

Categorical variables are reported as counts and percentages. Continuous data are presented as medians with interquartile ranges (IQR). Differences between proportions or medians were described using exact differences with 95% confidence intervals (CI).

We followed patients from the date of discharge from the index admission until the end of the follow-up period or until the time of readmission to hospital, emigration or death, whichever came first. Patients who died during the follow-up period without being readmitted, were censored on the time for death.

We used Cox regression to estimate hazard ratios (HRs) to predict the risk of readmission. First, HRs for readmission were estimated in a regression model only including age, gender, marital status and CCI. Second, we computed the HRs for readmission according to main reason for hospitalization (primary diagnosis) after adjustment for age, gender, marital status and CCI. Finally, in a model for the risk of readmission for male gender, we computed the HR after adjustment for age, CCI, marital status, and all primary diagnosis at index admission. The assumption of proportional hazards for all Cox regression models was assessed graphically using log(−log(survival probability))-plots.

Analyses were performed using the statistical software package Stata, version 15.1 (StataCorp, College Station, TX, USA).

## Results

### Baseline characteristics

A total of 93,306 patients aged 65 or older, were acutely admitted to Danish hospitals with a medical diagnosis and discharged within 24 h during the study period. The median age was 75 years (IQR 69 - 82), 49.5% were male, and 50.3% were married (Table 1). The proportion of patients with a CCI score of 1-2 and 3+ was 39.0 and 23.8%, respectively (Table 1). A history of cancer was relatively prominent in the two CCI groups, 35.5 and 64.5%, respectively.

**Table 1:** 30-day readmission according to baseline characteristics among older patients discharged early from hospitals

	Total Cohort^1^	Readmitted	Time to readmission, days, median (IQR)	Unchanged diagnosis^2^
Median age, years (IQR)	75 (69-82)	75 (70-82)	–	–
Age groups (years)				
65-75	44,467 (47.7%)	19.7%	8 (3-16)	30.8%
76-85	32,767 (35.1%)	21.1%	8 (3-17)	23.8%
86+	16,072 (17.2%)	20.4%	8 (3-16)	22.1%
Sex, n(%)				
Male	46,196 (49.5%)	22.2%	8 (3-16)	27.5%
Female	47,110 (50.5%)	18.5%	8 (3-16)	25.9%
Charlson Comorbidity Index				
0	34,672 (37.2%)	14.1%	7 (2-15)	30.2%
1-2	36,421 (39.0%)	20.4%	8 (3-16)	26.6%
3+	22,213 (23.8%)	30.0%	8 (3-17)	24.4%
Marital Status				
Married	46,965 (50.3%)	20.5%	8 (3-6)	28.5%
Not Married	46,330 (49.7%)	20.1%	8 (3-16)	25.0%
Unknown	11 (0.01%)	0.0%	–	–
Diseases of the cardiovascular system	13,038 (13.9%)	19.1%	9 (4-17)	45.3%
Angina Pectoris	1901 (2.0%)	17.7%	10 (4-18)	33.0%
Atrial fibrillation	7187 (7.7%)	19.3%	10 (4-18)	59.3%
Deep vein thrombosis	1324 (1.4%)	15.6%	6 (2-13)	16.4%
Hypertension	1622 (1.7%)	15.7%	7 (3-18)	21.7%
Heart failure	1004 (1.1%)	29.8%	8 (3-16)	34.1%
Diseases of the respiratory system	3653 (3.9%)	29.4%	9 (3-17)	37.8%
COPD	3088 (3.3%)	29.0%	9 (3-17)	42.4%
Respiratory failure	565 (0.6%)	31.5%	10 (2-18)	14.6%
Diseases of the nervous system	2091 (2.2%)	12.2%	7 (2-17)	26.8%
TIA	1441 (1.5%)	10.6%	6 (1-15)	24.2%
Epilepsy	650 (0.7%)	15.7%	11 (2-20)	29.4%
Endocrine and nutritional diseases	2765 (3.0%)	24.9%	7 (2-15)	21.5%
Diabetes	598 (0.6%)	22.4%	8 (2-17)	19.4%
Dehydration	2167 (2.3%)	25.5%	7 (3-14)	22.1%
Diseases of the digestive system	1962 (2.2%)	23.4%	6 (2-14)	19.4%
Dyspepsia	358 (0.4%)	15.6%	7.5 (3-14.5)	7.1%
Esophagitis	53 (0.1%)	17.0%	5 (1-14)	22.2%
Constipation	1502 (1.6%)	25.7%	5.5 (2-14)	21.5%
Ulcer without bleeding	49 (0.1%)	16.3%	4.5 (2-11)	0%
Hematological diseases				
Anemia	4168 (4.5%)	32.8%	12 (6-20)	42.5%
Infectious disease	7600 (8.1%)	23.2%	6 (2-14)	22.7%
Erysipelas	769 (0.8%)	21.9%	7 (3-17)	31.6%
Gastroenteritis	652 (0.7%)	21.8%	4 (1-13)	15.5%
Pneumonia	2711 (2.9%)	23.5%	6 (2-14)	28.7%
Urinary Tract Infection	3468 (3.7%)	23.6%	6 (2-14)	17.3%
Spinal disease/arthritis	1194 (1.3%)	17.7%	6 (3-13)	15.6%
Back Pain	1028 (1.1%)	19.0%	6 (2-12)	16.4%
Arthritis	166 (0.2%)	10.2%	7 (4-21)	5.9%
Mental and behavioral disorders				
Misuse of drugs or alcohol	1078 (1.2%)	17.2%	8 (2-15)	16.2%
R-diagnoses^3^	27,875 (29.8%)	21.4%	7 (3-15)	22.7%
Chest pain	4157 (4.5%)	15.8%	9 (4-19)	18.2%
Dyspnea	2450 (2.6%)	26.7%	8 (3-15)	18.7%
Fever	688 (0.7%)	32.4%	5 (2-14)	17.5%
Headache	569 (0.6%)	15.6%	9 (4-19)	5.6%
Pains in neuromuscular system	1035 (1.1%)	16.3%	7 (2-15)	6.5%
Abdominal pain	4920 (5.3%)	21.6%	6 (2-14)	24.9
Other pain	1247 (1.3%)	22.1%	7 (2.5-15)	11.2%
Vertigo	1986 (2.1%)	14.5%	7 (3-15)	16.0%
Other R-diagnoses	10,823 (11.6%)	23.5%	7 (2-15)	28.2%
Z-diagnoses^4^	24,392 (26.1%)	17.1%	8 (3-17)	18.9%
Malignancy	1526 (1.6%)	28.4%	10 (4-17)	18.4%
Central-nervous diseases	1841 (2.0%)	13.4%	7 (2-17)	8.5%
Heart diseases	3297 (3.5%)	15.8%	6 (1-14)	10.1%
Myocardial infarction	5705 (6.1%)	14.3%	10 (4-18)	21.0%
Rehabilitation	8037 (8.6%)	18.1%	7 (2-16)	25.0%
Other Z-diagnoses	3986 (4.3%)	17.7%	7 (2-16)	8.4%

*COPD* chronic obstructive pulmonary disease. *IQR* Interquartile range. *TIA* Transient ischaemic attack. ^1^Values reported are numbers (percentages of total cohort), unless otherwise stated

^2^Proportion of patients with readmission discharge diagnosis that did not differ from the diagnosis for the index discharge diagnosis. ^3^Used if symptoms, signs and abnormal clinical and laboratory findings are not elsewhere classified. ^4^Used when a diagnosis has not yet been confirmed

The most common discharge diagnoses were diseases of the cardiovascular system (13.9%), infectious diseases (8.1%), R-diagnoses (29.8%) and Z-diagnoses (26.1%) (Table 1).

### Mortality

A total of 2784 (3.0%) had died within the first 30 days after admission, and 1396 (1.5%) died without being readmitted.

### Readmission rate

Readmission rates, time to readmission and the proportion of patients readmitted with the same diagnosis as the index discharge diagnosis in relation to age, gender, marital status, comorbidity burden and the different discharge diagnoses are given in Table 1.

A total of 18,958 (20.3%; 95% CI 20.1 - 20.6) patients were readmitted within 30 days with a median time to readmission of 8 days (IQR 3 - 16). The time to readmission was shorter among patients with gastrointestinal, infectious, spinal/arthritis, and R discharge diagnoses and longer among patients with cardiovascular, respiratory, anemic discharge diagnoses and among patients where cancer suspicion was raised during the index admission (Table 1).

A total of 5067 (26.7%) readmitted patients had readmission discharge diagnoses that did not differ from the diagnosis for the index discharge diagnosis. The following groups had more often the same readmission discharge diagnosis as the index admission discharge diagnosis: youngest patients, male gender, low comorbidity burden, married, cardiovascular diseases (angina pectoris, atrial fibrillation, heart failure), chronic obstructive pulmonary disease (COPD), and anemia.

### Age, sex, marital status and comorbidity burden

In a regression model only including age, sex, marital status and CCI, we found that male sex (aHR 1.17, 95% CI 1.13-.1.20), CCI scores of 1-2 (aHR 1.49, 95% CI 1.43-1.54) and CCI scores 3+ (aHR 2.28, 95% CI 2.20-2.37) increased the risk of readmission within 30 days (Table 2). There was no association between being unmarried and the risk of readmission (Table 2). Adjusted HR of readmission according to the CCI score of 1-2 (aHR 1.41, 95% CI 1.35-1.46) or 3+ (2.13; 95% CI 2.03-2.23) was approximately constant after exclusion of patients (*n* = 18,221) with a history of malignant diseases.

**Table 2:** Unadjusted and adjusted hazard ratio for 30-day readmission according to age, sex, Charlson Comorbidity Index and marital status

	Unadjusted HR (95% CI)	Adjusted^2^ HR (95% CI)
Age^1^	1.03 (1.01-1.05)	1.01 (0.99-1.03)
Sex		
Female	Reference	Reference
Male	1.23 (1.19-1.26)	1.17 (1.13-1.20)
Charlson Comorbidity Index		
0	Reference	Reference
1-2	1.50 (1.44-1.55)	1.49 (1.43-1.54)
3+	2.33 (2.25-2.42)	2.28 (2.20-2.37)
Marital Status		
Married	Reference	Reference
Not Married	1.02 (1.00-1.05)	1.03 (1.00-1.07)

*CI* confidence interval; *HR* hazard ratio. ^1^Per 10-unit increase of variable ^2^Adjusted for the three other variables

### Discharge diagnoses

Table 3 shows aHRs for the risk of readmission according to the discharge diagnoses. Patients admitted with heart failure, COPD, respiratory failure, dehydration, constipation, anemia, pneumonia and urinary tract infection had an increased risk of being readmitted within 30 days after discharge. Furthermore, the R-diagnoses dyspnea, fever, abdominal pain, other R-diagnoses and a Z-diagnosis of malignancy increased the risk of readmission (Table 3). Several diagnoses of the cardiovascular system (angina pectoris, deep vein thrombosis, hypertension and transient ischemic attack) were associated with a reduced risk of readmission. Arthritis, epilepsy, pain in the neuromuscular system, headache, chest pain, vertigo and Z-diagnoses related to the central nervous system and heart diseases, were also associated with a reduced risk of readmission (Table 3).

**Table 3:** Unadjusted and adjusted hazard ratios for 30-day readmission among older patients discharged early from emergency departments

	Unadjusted HR (95% CI)	Adjusted HR (95% CI)^1^
Diseases of the cardiovascular system		
Angina Pectoris	0.84 (0.76-0.94)	0.83 (0.75-0.93)
Atrial fibrillation	0.93 (0.88-0.98)	1.05 (1.00-1.11)
Deep vein thrombosis	0.75 (0.65-0.86)	0.82 (0.71-0.94)
Hypertension	0.74 (0.66-0.84)	0.86 (0.76-0.97)
Heart failure	1.56 (1.39-1.75)	1.26 (1.12-1.41)
Diseases of the respiratory system		
COPD	1.51 (1.41-1.62)	1.33 (1.25-1.43)
Respiratory failure	1.74 (1.51-2.02)	1.55 (1.34-1.79)
Diseases of the nervous system		
TIA	0.49 (0.42-0.58)	0.54 (0.46-0.63)
Epilepsy	0.74 (0.61-0.90)	0.71 (0.59-0.86)
Endocrine and nutritional diseases		
Diabetes	1.12 (0.95-1.33)	0.90 (0.76-1.07)
Dehydration	1.35 (1.24-1.47)	1.28 (1.17-1.39)
Diseases of the digestive system		
Dyspepsia	0.75 (0.57-0.97)	0.80 (0.62-1.04)
Esophagitis	0.82 (0.43-1.58)	0.81 (0.42-1.55)
Constipation	1.34 (1.20-1.47)	1.26 (1.14-1.39)
Ulcer without bleeding	0.80 (0.40-1.59)	0.77 (0.39-1.55)
Hematological diseases		
Anemia	1.72 (1.63-1.82)	1.45 (1.38-1.54)
Infectious disease		
Erysipelas	1.08 (0.93-1.26)	1.08 (0.93-1.25)
Gastroenteritis	1.10 (0.93-1.30)	1.10 (0.93-1.29)
Pneumonia	1.22 (1.13-1.32)	1.15 (1.06-1.25)
Urinary Tract Infection	1.21 (1.13-1.30)	1.15 (1.07-1.24)
Spinal disease/arthritis		
Back Pain	0.93 (0.81-1.07)	1.02 (0.88-1.17)
Arthritis	0.47 (0.29-0.76)	0.51 (0.31-0.81)
Mental and behavioral disorders		
Misuse of drugs and alcohol	0.83 (0.72-0.96)	0.85 (0.74-0.99)
R-diagnoses^2^		
Chest pain	0.74 (0.68-0.80)	0.76 (0.70-0.82)
Dyspnea	1.38 (1.28-1.49)	1.25 (1.15-1.35)
Fever	1.77 (1.55-2.02)	1.52 (1.33-1.73)
Headache	0.74 (0.60-0.91)	0.80 (0.65-0.99)
Pains in neuromuscular system	0.79 (0.68-0.92)	0.80 (0.69-0.94)
Abdominal pain	1.08 (1.02-1.15)	1.12 (1.05-1.19)
Other pain	1.11 (0.99-1.25)	1.09 (0.96-1.22)
Vertigo	0.68 (0.61-0.77)	0.73 (0.65-0.82)
Other R-diagnoses	1.21 (1.16-1.27)	1.18 (1.13-1.23)
Z-diagnoses^3^		
Malignancy	1.46 (1.32-1.60)	1.51 (1.37-1.66)
Central-nervous diseases	0.63 (0.55-0.71)	0.66 (0.58-0.75)
Heart diseases	0.75 (0.69-0.82)	0.81 (0.74-0.88)
Myocardial infarction	0.66 (0.61-0.70)	0.67 (0.62-0.72)
Rehabilitation	0.87 (0.83-0.92)	0.88 (0.84-0.93)
Other Z-diagnoses	0.86 (0.79-0.93)	0.88 (0.82-0.95)

*CI* confidence interval. *COPD* chronic obstructive pulmonary disease. *HR* hazard ratio. *TIA* transient ischaemic attack

^1^Adjusted for age, gender, Charlson Comorbidity Index and marital status

^2^Used if symptoms, signs and abnormal clinical and laboratory findings are not elsewhere classified. ^3^ Used when a diagnosis has not yet been confirmed

### Male gender and readmission

The association between male gender and the risk of readmission was analyzed in an additional regression model where all other variables were included in the model. Also, in that model male gender (aHR 1.15, 95% CI 1.11-1.18) was associated with an increased risk of readmission. There was no difference in time to readmission between male and females. Males were slightly more likely than females to be readmitted and discharged with the same diagnosis as the index discharge diagnosis (27.5% vs 25.9, 95% CI of the difference 0.3-2.9).

## Discussion

Our knowledge of unplanned readmission rates and risk factors for readmission among older people after early discharge from ED is sparse. To the authors knowledge, this is the first national study of acutely admitted patients aged 65+ years and discharged within 24 h. One fifth were readmitted unplanned within 30 days. Male sex, a high burden of comorbidity and a wide range of primary discharge diagnoses and symptoms were identified as risk factors for readmission. A total 26.7% readmitted patients had readmission discharge diagnoses that did not differ from the diagnosis for the index discharge diagnosis.

### Readmission rate

There exist no comparable data on readmission rates following early discharge of acutely admitted older people. Studies of older patients with a longer duration of hospital stay have shown 30-day readmission rates from 13 to 22% [9–13]. It is noteworthy that 29.8% of the patients with heart failure and 23.5% of the patients with pneumonia in our study were readmitted within 30 days (Table 1). This is slightly higher than the previous published readmission rates for older patients with the same discharge diagnoses even after a longer duration of hospital stay [11–13, 20].

A clinical decision that discharge within 24 h after arrival was possible may hypothetically indicate the presence of non-serious acute diseases and fewer untreated chronic medical problems among the patients in our study. However, it is not possible to reveal any conclusions about that based on our data. Although we cannot account for the many other and very complex conditions [21] that may have increased the risk of re-admission of the patients in our study, it cannot be ruled out that the early discharge may have left unresolved problems [22, 23] that have led to an increased risk of re-admission and a higher readmission rate than expected for some diseases in our study.

### Age, sex and marital status

Some studies have shown that older people have an increased risk of being readmitted [12, 24, 25]. However, our data did not confirm an increased risk of readmission among the oldest age groups. The oldest age group was more frequently readmitted with a new clinical problem than the index admission, which could be explained by an increased comorbidity burden and an increased risk for a new clinical problem to arise [25]. We cannot report details on the medical, social or institutional support to the patients after discharge, but optimized discharge procedures and discharge to nursing care facilities among the very old patients may have contributed to our findings of equal admission rates in the different age groups.

The men in our study had a higher risk of readmission than the women, as observed in most [12, 24–26], but not all [9, 27] earlier studies. Additionally, men were more commonly readmitted with the same diagnosis as the index discharge diagnosis. There are no clear-cut explanations for the findings that men had a higher re-admission rate than women. Others [25, 28] have suggested that differences in health-seeking behaviors and adherence to treatment between men and women may to some extent explain the gender differences in the risk of readmission. However, further prospective studies including medical conditions, adherence to treatment, and compliance with follow-up and health recommendations [12] are needed to interpret the differences in readmission rate and further to design studies of the impact of specific interventions targeting men.

We were not able to obtain information on single-living status, which is considered a proxy for social isolation and has been shown to increase the risk of readmission and mortality [29]. Information about marital status was the best proxy available in our study, but we did not find any association between unmarried older patients and risk of readmission.

### Comorbidity and discharge diagnoses

We observed that the risk of readmission increased with greater comorbidity burden. These findings are in line with previously published results [12, 24, 25, 27, 30, 31]. Patients with a comorbidity burden of CCI3+ had twice the risk of readmission compared with patients without comorbidities even after exclusion of the large group of patients with previously diagnosed malignant diseases. The proportion of readmitted patients who were discharged with a different diagnosis than the index admission increased with increasing comorbidity burden which is consistent with previous research [25]. Increasing number of comorbid conditions increases the risk of a new clinical problem to arise after early discharge. When considering interventions to mitigate readmissions it might be reasonable to suggest that older patients with a high comorbidity burden should be identified prior to early discharge to ensure an optimal management and follow-up of the comorbidities.

The results from our study are consistent with other studies [10, 12, 20, 25, 30, 32], in that the risk of readmission was associated to a wide range of specific diseases and discharge diagnoses including heart failure, COPD, dehydration, constipation, anemia, pneumonia and urinary tract infection. Furthermore, a large group of patients were discharged with a symptom diagnosis related to the ICD-10 R-code classification rather than a specific disease code and among these dyspnea, fever, abdominal pain and a mixed group of other R diagnoses were associated with an increased risk of unplanned acute readmissions. The relatively high prevalence of the non-specific diagnoses in our study has also been reported in another Danish study [33] of 264,265 acute medical patients admitted to all hospitals in Denmark. Almost one fifth of the patients with R and Z diagnoses during index admission were discharged after readmission with the same unspecific R- or Z-diagnosis. In a prevention perspective, it might be relevant to study more thoroughly whether some of the readmissions among early discharged patients could be avoided if a more complete workup of the patients’ symptoms could reveal a more accurate diagnosis, provide better managements and thereby reducing the risk of readmission.

Our study clearly revealed that the majority of readmissions are due to a different clinical problem than the index admission, making it difficult to predict the risk of re-admission and to establish interventions based on the discharge diagnosis. However, our data also showed that half the patients had been readmitted by day 8. It has been shown in a group of younger general medical patients (mean age 55 years) with a mean LOS of 5-6 days that early readmissions are more likely to be preventable and amenable to hospital-based interventions [23]. Readmissions in the week after discharge were more likely to be caused by factors (premature discharge, problems with physician decisions related to diagnosis and treatment) over which the hospital has direct control compared to later readmissions [23]. We do not have effective and validated tools to screen older patients for suitability for early discharge. Although we cannot conclude from our data on the quality of the discharge process and whether it was optimal in avoiding re-admission, our and others findings [23] could be a direction for further research in identifying older patients at increased risk for post discharge problems and readmission after early discharge.

There is a lack of prospective analysis and validation of prediction models that can identify older patients at increased risk of readmission after early discharge. Male gender, comorbid conditions, patients with unspecific diagnoses due to incomplete work-up during admission, and the discharge diagnoses associated to increased risk of readmission in our study, could be important characteristics that require attention from the emergency department staff before discharge, and may also be target interventions in future readmission research.

### Factors associated with lower risk of readmission

Angina pectoris, deep vein thrombosis, hypertension and transient ischemic attack, arthritis, epilepsy, pain in the neuromuscular system, headache, chest pain, vertigo and Z-diagnoses related to the central nervous system and heart diseases, were associated with a reduced risk of readmission. It is not possible to describe the post-discharge care among our patients and the potential impact on the risk of readmission. However, planned ambulatory follow-up and further monitoring by GPs after the discharge could partly explain the reduced risk of re-admission among these patient groups.

### Implications

Our study can be used to shed light on the risk of readmission among older people after early discharge from emergency departments. We have identified older patients at increased risk of readmission. Although the results should be interpreted in the light of study limitations our results can be helpful in developing readmission procedures among older patients and to perform research in interventions to reduce the risk of readmission after early discharge. Transitional care intervention programs delivered to older patients by a discharge nurse coach during hospital stay and after discharge have shown reduced readmissions [34–36]. We do not have any data on the effect of transitional care intervention programs among acutely admitted older medical patients who are discharged early. However, following our findings we may suggest that transitional care programs should have increased attention to older men, patients with high comorbidity burden, patients with non-specific discharge diagnoses and some specific discharge diagnoses such as heart failure and pneumonia.

### Strengths and limitations

This study has important strengths. Our database contains all emergency medical admissions in Denmark over a period of 18 months and we have used national registries with complete follow-up for readmission and mortality thereby reducing the risk of selection bias. The database is population-based and includes patients from a uniform tax-supported healthcare system, which reduces the risk of referral bias. The number of included patients was high, which minimizes the risk of random errors. The study has several limitations. We have analyzed the potential confounding caused by age, gender, comorbidity burden and marital status in our estimates of associations between exposing variables and readmission. However, our estimates may have been biased by unmeasured confounding. We did not have access to data regarding socio-economic status [37] medication [22, 38], functional impairment [22, 31], social support [39], and whether patients were discharged to their own homes or nursing homes. In may be relevant to include these factors in further research.

More than half of the patients were diagnosed with non-specific R- and Z-diagnoses. This high prevalence was in line with other studies [33]. These findings may possibly increase the risk of misclassification and bias in our findings. We have analyzed the total comorbidity burden of the patients by use of a national registry (DNRP). The positive predictive values of the coding’s in the DNRP is high [19] However, we cannot rule out underestimation of comorbidity burden and thereby unmeasured confounding.

## Conclusions

One fifth of the acutely admitted medical older patients discharged within 24 h from hospitals were readmitted within 30 days. Male sex, the burden of comorbidity and several primary discharge diagnoses were risk factors for readmission. Most of the readmissions were due to a new diagnosis especially among patients with comorbid conditions. However, these findings should be interpreted in the light of the large proportion of patients who were discharged with non-specific diagnoses.

## Data Availability

Data are stored by Aarhus University Hospital, Denmark, on servers with security according to the rules given by Danish law. The data can be made available by reasonable request to the corresponding author.

## Abbreviations

aHR: Adjusted hazard ratios
CCI: Charlson Comorbidity Index
CI: Confidence interval
COPD: Chronic obstructive pulmonary disease
CRS: Danish Civil Registration System
DNRP: Danish National Registry of Patients
ED: Emergency Department
GP: General practitioner
ICD: International Classification of Diseases
IQR: Interquartile range
LOS: Length of stay
